# Immunization with the MipA, Skp, or ETEC_2479 Antigens Confers Protection against Enterotoxigenic *E. coli* Strains Expressing Different Colonization Factors in a Mouse Pulmonary Challenge Model

**DOI:** 10.3389/fcimb.2016.00181

**Published:** 2016-12-12

**Authors:** Michael P. Hays, Amit Kumar, Francisco J. Martinez-Becerra, Philip R. Hardwidge

**Affiliations:** ^1^College of Veterinary Medicine, Kansas State UniversityManhattan, KS, USA; ^2^Immunology Core Laboratory of the Kansas Vaccine Institute and Department of Pharmaceutical Chemistry, University of KansasLawrence, KS, USA

**Keywords:** ETEC, vaccines, antigens, intranasal immunization, colonization factor

## Abstract

Achieving cross-protective efficacy against multiple bacterial strains or serotypes is an important goal of vaccine design. Enterotoxigenic *Escherichia coli* (ETEC) is an important cause of diarrheal disease in underdeveloped nations. We have been interested in identifying and characterizing ETEC antigens that generate protective immune responses independent of ETEC colonization factor (CF) expression. Our previous studies used proteomics to identify the ETEC MipA, Skp, and ETEC_2479 proteins as effective in protecting mice from homologous challenge with ETEC H10407 using a pulmonary inoculation model. This model permits analysis of mouse survival, bacterial clearance, and the production of secretory IgA (sIgA) and has been employed previously for studies of enteric pathogens for which robust oral challenge models do not exist. MipA belongs to a family of proteins involved in remodeling peptidoglycan. Skp rescues misdirected outer membrane proteins. ETEC_2479 is predicted to function as an outer membrane porin. These proteins are conserved in pathogenic ETEC strains as well as in commensal *Proteobacteria*. Antibodies produced against the ETEC MipA, Skp, and ETEC_2479 proteins also reduced the adherence of multiple ETEC strains differing in CF type to intestinal epithelial cells. Here we characterized the ability of 10 heterologous ETEC strains that differ in CF type to cause clinical signs of illness in mice after pulmonary challenge. ETEC strains C350C1A, E24377A, E7476A, WS2173A, and PE360 caused variable degrees of lethality in this mouse model, while ETEC strains B7A, WS6866B, 2230, ARG-2, and 8786 did not. Subsequent challenge experiments in which mice were first vaccinated intranasally with MipA, Skp, or ETEC_2479, when combined with cholera toxin, showed both that each antigen was protective and that protection was strongly correlated with fecal IgA concentrations. We conclude that the MipA, Skp, or ETEC_2479 antigens generate protection in the mouse pulmonary challenge model against ETEC strains that express different CFs.

## Introduction

Enterotoxigenic *Escherichia coli* (ETEC) continues to be a health scourge both to endemic populations living in underdeveloped countries, especially children, as well as to vacationers and military personnel that travel to these regions (Fleckenstein et al., 2010). We have been interested in identifying and characterizing ETEC proteins that might serve as potential vaccine targets (Kumar et al., 2015; Hays et al., 2016). Many previous vaccine strategies have focused on heterogeneous surface structures known as colonization factors (CFs; Fleckenstein et al., 2014). However, given the diversity of CFs, identification of additional antigens may improve the cross-protective efficacy of future vaccine formulations.

We have been characterizing the potential protective efficacy of the ETEC MipA, Skp, and ETEC_2479 proteins in a pulmonary challenge model (Kumar et al., 2015). We focused on these antigens after performing proteomic studies of ETEC H10407 (Kumar et al., 2015). MipA belongs to a family of proteins involved in remodeling peptidoglycan. Skp rescues misdirected outer membrane proteins. ETEC_2479 is predicted to function as an outer membrane porin (Kumar et al., 2015). While the potential role of these proteins in ETEC virulence is not apparent, it is known that these proteins are conserved among pathogenic and non-pathogenic *E. coli* (Kumar et al., 2015; Hays et al., 2016). It is also known that MipA can be detected in immunoblots using sera from mice and humans (Roy et al., 2010).

The pulmonary challenge model permits analysis of mouse survival, bacterial clearance, and the production of secretory IgA (sIgA) (van de Verg et al., 1995; Turbyfill et al., 2000) and has been employed previously for studies of enteric pathogens for which robust oral challenge models do not exist including the analysis of pathogenicity and immune responses to several ETEC strains (Byrd and Cassels, 2003). We found previously that immunizing mice with MipA, Skp, and ETEC_2479 was protective against homologous challenge with ETEC H1407 (Kumar et al., 2015). Furthermore, despite the conservation of these antigens among Gram-negative bacteria, mouse health was not negatively impacted nor were significant alterations to the mouse intestinal microbiota observed as a function of vaccination (Hays et al., 2016). Antibodies raised against the MipA, Skp, and ETEC_2479 antigens also reduced the *in vitro* cell adherence of a panel of heterologous ETEC strains (Kumar et al., 2015). Here we determined the extent to which vaccination with MipA, Skp, or ETEC_2479 would protect mice challenged intranasally with a panel of diverse ETEC strains differing in CF type.

## Materials and methods

### Ethics statement

The Kansas State University Institutional Animal Care and Use Committee approved the animal procedures (IACUC protocol #3196) in the context of the Kansas State University Animal Welfare Assurance Number A3609-01, in compliance with the Public Health Service (PHS) Policy on Humane Care and Use of Laboratory Animals.

### Bacterial strains and infections

The ETEC strains used are described in Table 1. Female BALB/c mice (3 weeks old) were obtained from the Jackson Laboratory (Bar Harbor, Maine), housed in microisolator cages, and provided with food and water *ad libitum*.

**Table 1:** **ETEC strains used in this study**.

**Strain**	**CF type**	**Toxin(s)**	**Serotype**	**Location**	**Reference**	**MipA**	**Skp**	**ETEC_2749**
H10407	CFA/I	LT, STh-STp	O78:H11	Bangladesh	Evans and Evans, 1973	+	+	+
E24377A	CS1, CS3	LT, ST	O139:H28	Egypt	Tacket et al., 1994	+	+	+
B7A	CS6	LT, ST	O148:H28	Vietnam	DuPont et al., 1971	+	+	+
WS6866B	CS8	LT	O25:H-	Egypt	Shaheen et al., 2004	+	+	+
2230	CS10	LT, STp	025:H16	Senegal	Darfeuille-Michaud et al., 1986	+	+	+
350C1A	CS12	LT, STp	O159:H4	Kenya	Levine et al., 1983	+	+	+
PE360	CS13	LT	O9:H-	Australia	Heuzenroeder et al., 1990	+	+	+
E7476A	CS14	STh	O166:H27	South Africa	McConnell et al., 1989	+	+	+
8786	CS15	–	O117:H4	Burundi	S. Savarino	+	+	+
ARG-2	CS18	LT, STp	O20:K27:H-	Argentina	Viboud et al., 1993	+	+	+
WS2173A	CS23	LT	O71:H4	Egypt	S. Savarino	+	+	+

We first determined the ability of 10 ETEC strains other than H10407 to induce clinical signs of illness in a pulmonary challenge model. ETEC strains were cultivated overnight on CFA agar plates, resuspended in sterile phosphate-buffered saline (PBS) and diluted to an OD_600_ of 1.0 [~1 ^*^ 10 colony forming units (CFUs)/ml; (Byrd and Cassels, 2003)]. Mice were lightly anesthetized with isoflurane and challenged intranasally with 5 ^*^ 10^8^ CFUs of individual ETEC strains by dropwise administration of 50 μl of the ETEC suspensions to the external nares of each mouse. Mice were observed every 4 h after challenge and clinical signs of illness (lack of responsiveness to stimulation, hunched posture, ruffled hair coat, dehydration) were recorded. If mice displayed clinical signs of illness, or at the end of the study (7 d), they were euthanized, necropsied, and their lungs were removed aseptically. Lungs were homogenized, serially diluted in PBS, and plated on MacConkey agar to enumerate ETEC.

For vaccination studies, antigens were administered intranasally at 20 μg/dose with 2.5 μg of cholera toxin (Sigma-Aldrich) in 25 μl PBS to the external nares of mice that had been lightly anesthetized with isoflurane. Booster doses were administered 2- and 4-weeks after the initial vaccination. Mice were then challenged with ETEC strains as described above. The antigens used in this study were purified and prepared as glutathione-S-transferase (GST)-fusion proteins as described previously (Kumar et al., 2015; Hays et al., 2016). A GST epitope control protein was also used as a negative control for immunization studies.

### Immunoassays

IgA concentrations in mouse feces were quantified using ELISAs. Five fresh stool pellets from each animal were added to 1 ml of fecal reconstitution buffer (50 mM ethylenediaminetetraacetic acid (EDTA), 0.1 mg/ml soybean trypsin inhibitor, 1.39 μg/ml phenylmethylsulfonylfluoride (PMSF), and homogenized. Samples were centrifuged (5 min, 5000 g) and supernatants (50 μl) were added to polystyrene 96-well, flat bottom plates (Whatman) that had coated with 0.5 μg/ml of each purified protein or BSA. After overnight incubation, a rabbit anti-mouse IgA HRP detection antibody (Sigma) diluted 1:4000 in 0.1% PBS-Tween was added. Plates were developed with 1-StepTM Ultra TMB-ELISA (Thermo) and quenched with 3 N H_2_SO_4_. Absorbance was read at 450 nm.

### Statistical analyses

Differences in mouse survival as a function of time after ETEC challenge were analyzed using Log-rank tests. Differences in both ETEC loads in mouse lungs and in fecal IgA concentrations were analyzed using Kruskal-Wallis tests. Asterisks indicate significant differences at *p* < 0.05.

## Results

We previously reported the efficacy of immunizing mice with recombinant forms of the ETEC H10407 Skp, MipA, and ETEC_2479 proteins in protecting mice against an otherwise lethal challenge with ETEC H10407 (Kumar et al., 2015). We had also shown previously that antibodies raised against the Skp, MipA, and ETEC_2479 proteins were able to protect cultured intestinal epithelial cells from adherence by these ETEC strains (Kumar et al., 2015). Here we desired to determine the extent to which immunizing mice with these antigens might confer protection toward heterologous ETEC strains that differ in CF type.

We infected 11 separate groups of mice (*n* = 5 group) with 10 different ETEC strains (5 ^*^ 10^8^ CFUs), as well as with ETEC H10407 as a positive control, and evaluated the extent to which they induced clinical signs of illness in the mice meriting euthanasia. These strains were chosen for their diversities in CF type, geographical points of isolation, and toxins (Table 1).

**Figure 1:**
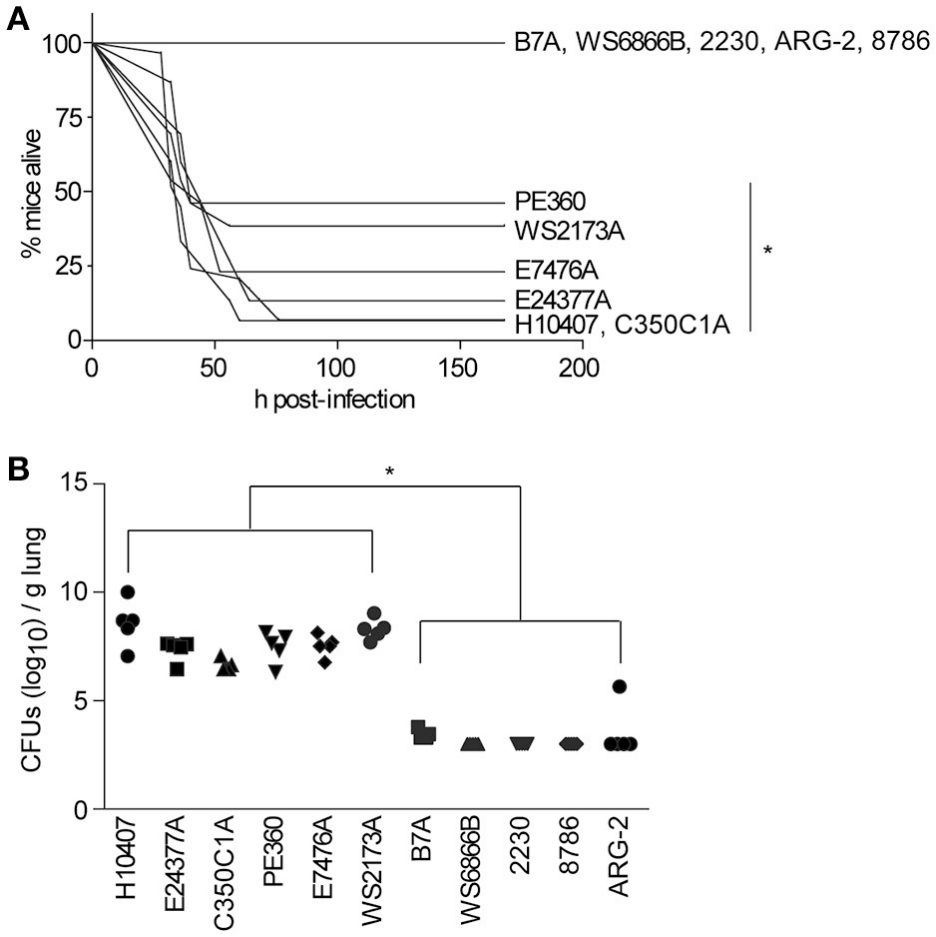
**ETEC strains that cause illness in the mouse pulmonary challenge model. (A)** Mouse survival is plotted as a function of time (h) after mice were inoculated with the indicated ETEC strains. H10407, *n* = 29; 350C1A and E24377A, *n* = 15; E7476A, WS2173A, and PE360 *n* = 13; B7A, WS6866B, 2230, ARG-2, and 8786, *n* = 5/group. Asterisks indicate significantly different (*p* < 0.05) mouse survival, log-rank test. **(B)** ETEC loads (CFUs/g lung) in mice infected with the indicated ETEC strains at time of euthanasia or at the end of the study (7 d), *n* = 5/group. Asterisks indicate significantly different (*p* < 0.05) ETEC loads in mouse lungs, Kruskal-Wallis test.

We observed that, in addition to H10407 (27/29 mice), C350C1A (14/15 mice) caused extensive amounts of lethality, with median survival times of 36 h (Figure 1A). E24377A, E7476A, WS2173A, and PE360 yielded intermediate phenotypes, causing lethality in 13/15, 10/13, 8/15, and 7/13 mice, respectively, with median survival times of 44, 44, 40, and 40 h (Figure 1A). ETEC strains B7A, WS6866B, 2230, ARG-2, and 8786 caused no lethality (0/5 mice).

We also quantified the amounts of ETEC strains present in the mouse lungs at the time of euthanasia. ETEC loads were relatively high in mouse lungs in which infection caused lethality, while they were relatively low in mouse lungs in which infections were non-lethal (Figure 1B; *p* < 0.05, Kruskal-Wallis test). Given these data, we therefore pursued vaccination studies with the strains PE360, WS2173A, E7476A, E24377A, and C350C1A.

**Figure 2:**
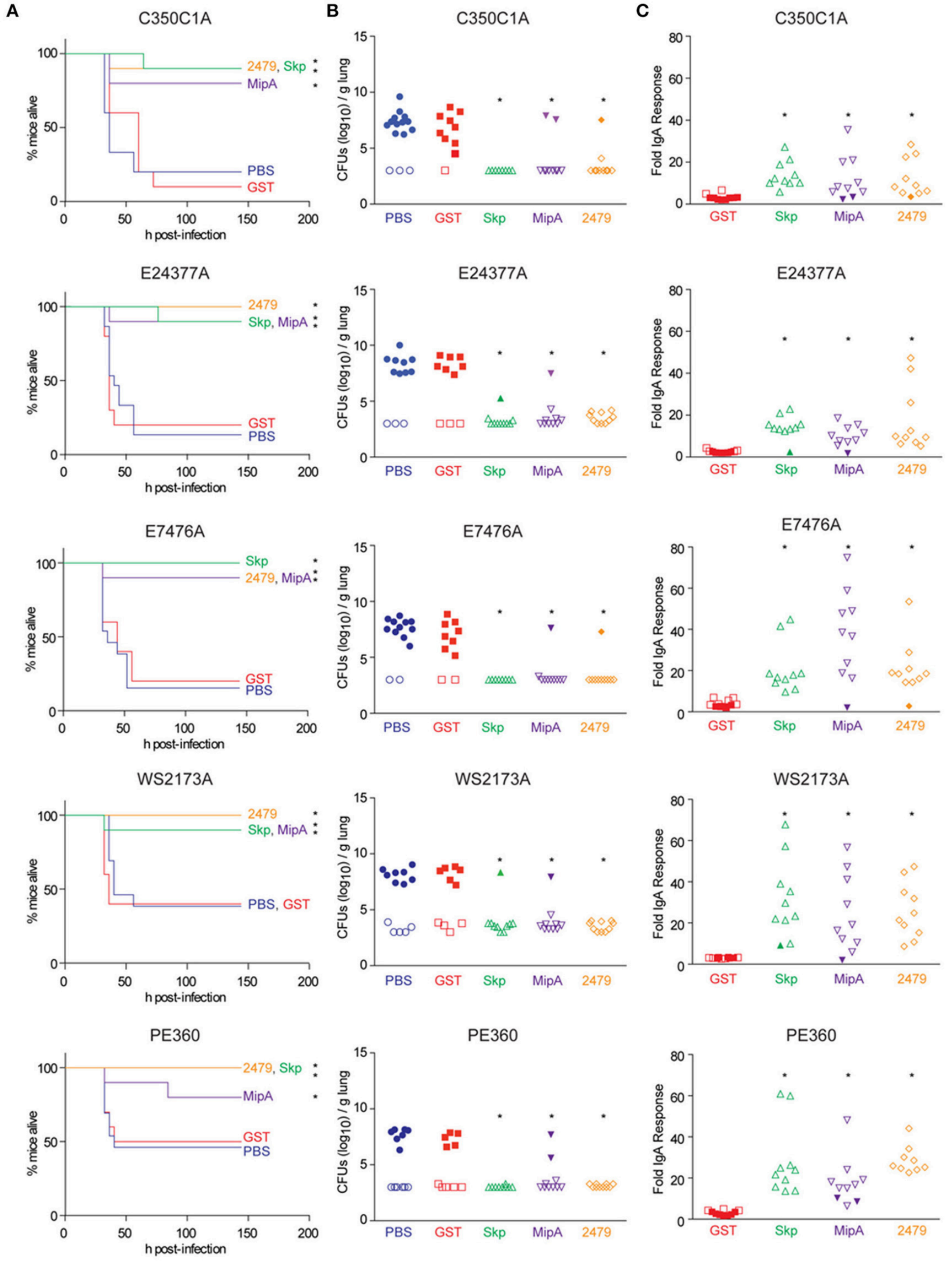
**Impact of vaccination on mouse survival after pulmonary challenge with ETEC. (A)** Mouse survival is plotted as a function of time (h) after mice were inoculated with the indicated ETEC strains following intranasal immunization with the indicated antigens, *n* = 10–15. Asterisks indicate significantly different (*p* < 0.05) mouse survival, log-rank test. **(B)** ETEC loads (CFUs/g lung) in mice infected with the indicated ETEC strains at time of euthanasia or at the end of the study (7 d). Open symbols indicate mice that survived for the duration of the study. Closed symbols indicate mice that were euthanized due to their display of clinical signs of illness, *n* = 10–15. Asterisks indicate significantly different (*p* < 0.05) ETEC loads in mouse lungs, Kruskal-Wallis test. **(C)** Fold change in mouse fecal IgA concentrations after immunization with the indicated antigens. Open symbols indicate mice that survived for the duration of the study. Closed symbols indicate mice that were euthanized due to their display of clinical signs of illness. *n* = 10. Asterisks indicate significantly different (*p* < 0.05) fecal IgA concentrations, Kruskal-Wallis test.

Mice were immunized three times at 2-week intervals with individual antigens combined with cholera toxin. Mice were then inoculated intranasally with individual ETEC strains and evaluated for clinical signs of disease over a 7-day period. All three antigens were protective against the infectious challenge, regardless of the ETEC strain (Figure 2A). Whereas mice vaccinated with either PBS or a GST-epitope control protein succumbed to infection at similar rates and frequencies as shown in initial studies (Figure 1A), mice vaccinated with either Skp, MipA, or ETEC_2479 were generally protected from ETEC challenge, with only 1 or 2/10 non-responding mice in each group (Figure 2A, Table 2; *p* < 0.05, Log-rank test).

**Table 2:** **Pulmonary challenge data**.

**Strain**	**Median survival (h)^a^**	**Survival rate**				
		**(# survived/# challenged) after challenge in vaccinated mice**				
		**PBS**	**GST**	**Skp**	**MipA**	**2479**
H10407	36	2/29^b^	2/20^b^	17/25^b^	16/25^b^	22/25^b^
350C1A	36	3/15	1/10	9/10	8/10	9/10
PE360	40	6/13	5/10	10/10	8/10	10/10
WS2173A	40	5/13	4/10	9/10	9/10	10/10
E7476A	44	2/13	2/10	10/10	9/10	9/10
E24377A	44	2/15	2/10	9/10	9/10	10/10

a No lethality was observed for ETEC B7A, WS6866B, 2230, 8786, and ARG-2 when administered at a dose of 5 _*_ 10^8^ CFUs, so these strains were not used in vaccination studies.

b *Data were previously described in Kumar et al. (2015)*.

We observed high loads of ETEC (~10^6−10^ CFUs/g) in the lungs of mice that were euthanized due to their presentation of clinical signs of disease (Figure 2B). By contrast, relatively little ETEC was cultured from the lungs of mice that survived the infection (Figure 2B). ETEC loads were inversely related to fecal IgA concentrations in mice (Figure 2C). Fecal IgA responses were significantly correlated with mouse survival, as mice that did not develop significant fecal IgA responses against the antigens did not survive the infectious challenge (Figure 2C). The results described here support our previous findings that characterized the ability of antisera raised against Skp, MipA, or ETEC_2479 to protect against the adherence of the strains described here to intestinal epithelial cells (Kumar et al., 2015).

## Discussion

We have established here that immunizing mice with the Skp, MipA, or ETEC_2479 antigens protects mice not only against challenge with ETEC H10407, but also against challenge with the ETEC strains PE360, WS2173A, E7476A, E24377A, and C350C1A. The ability of different ETEC strains to cause disease in mice using the pulmonary challenge model appeared to be unrelated to toxin type, as strains encoding LT and/or ST were equally distributed among strains that did or did not cause disease. It is unclear why some strains cause illness in this model and others do not. This topic could be addressed in the future by conducting comparative genome analyses to identify virulence determinants.

While Byrd et al. previously observed that challenge of BALB/c mice with 5 ^*^ 10^8^ CFUs of ETEC B7A caused mortality in 25% (3/12) mice (Byrd and Cassels, 2003), we did not observe any mortality at this dose. A limitation of our study is that we conducted all challenge assays using a single dose (for direct comparison to our previous studies of ETEC H10407), rather than performing dose-finding assays. It is conceivable that higher doses of B7A, WS6866B, 2230, ARG-2, and 8786 could cause mouse mortality in this challenge model. The ability of some ETEC strains to colonize mice in this model may also be related to their different CF types.

While the pulmonary challenge model is useful in the preliminary assessment of vaccine antigens in the study of enteric pathogens for which robust oral challenge models do not exist (Byrd and Cassels, 2003), there are several significant limitations to this model. These limitations include the lack of diarrhea, potential differences in ETEC receptors between lung and intestinal tissue, and differing microbiomes and mucosal interfaces. Our use of CT as an adjuvant for vaccination also limits the potential clinical relevance of our data.

As expected, surviving animals consistently had lower CFU counts in the lungs. Differences in IgA responses among mice matched the protection patterns across vaccinated animals. These data suggest that mucosal immunity, and in particular, levels of specific IgA might play a role in protection. Opsonization and/or direct anti-microbial activity might also play a role during infection (Eyles et al., 1998). Further studies will define the particular role of IgA in this model. We did not observe significant differences in cytokine responses when we analyzed lung homogenates using a mouse TH1/TH2 9-Plex Tissue Culture Kit (Meso Scale Discovery; data not shown). We plan in subsequent experiments to evaluate the extent to which these antigens may have cross-protective efficacy if used with adjuvants other than cholera toxin and/or in other routes of administration.

## Author contributions

MH and AK performed the experiments. PH designed the study. MH, FM, and PH analyzed the data and wrote the manuscript.

### Conflict of interest statement

The authors declare that the research was conducted in the absence of any commercial or financial relationships that could be construed as a potential conflict of interest.
